# Self-inflicted penetrating chest trauma from solar powered garden light: a case report

**DOI:** 10.1093/jscr/rjab320

**Published:** 2021-08-09

**Authors:** Lewis William Murray, Jayme Bennetts

**Affiliations:** Department of Cardiothoracic Surgery, Flinders Medical Centre, Adelaide, South Australia, Australia; Department of Cardiothoracic Surgery, Flinders Medical Centre, Adelaide, South Australia, Australia; College of Medicine and Public Health, Flinders University, Adelaide, South Australia, Australia

## Abstract

Penetrating chest trauma is associated with significant morbidity and mortality due to direct injury to vital organs located within the thorax. This is a case of a 53-year-old man who presented with a self-inflicted penetrating chest trauma using a solar powered garden light. The light penetrated the left side of his chest resulting in a haemopneumothorax, diaphragmatic perforation and pericardial haematoma. The patient underwent an urgent explorative thoracotomy for the removal of the garden light, repair of the diaphragmatic perforation and wedge resections of the perforated lung parenchyma. Postoperatively, the patient recovered in the intensive care before being transferred to the psychiatric department.

## INTRODUCTION

Injuries secondary to chest trauma are associated with significant morbidity and mortality. Chest trauma is typically divided into two groups: the first being from blunt force trauma and the second being from penetrating trauma. Penetrating chest injuries are due to either high-energy injuries, like gunshots or low energy injuries like stabbings.

For individuals who survive the immediate penetrating injury, life threating complications can develop like pneumothorax, cardiac tamponade and massive haemorrhage. These conditions require immediate interventions until a patient can receive definitive surgical treatment.

We present a case of self-inflicted penetrating thoracic trauma with an unusual object.

## CASE REPORT

This case report describes a 53-year-old man who presented as a Level 1 trauma immediately after intentionally stabbing himself in the left side of the chest with a solar powered garden light. His medical history was significant for prior self-inflicted penetrating abdominal injury, tetrahydrocannabinol (THC) misuse, hepatitis B and schizoaffective disorder.

On arrival, there was a metal rod protruding from the left anterior chest at the level of the fourth rib anteriorly, which moved in a rhythmic manner consistent with his electrocardiogram (ECG) tracing. Despite this, the patient was able to maintain his own airway, had normal oxygen saturations on room air, was haemodynamically stable and had a glascow coma score (GCS) of 14. There was no ‘sucking’ around the foreign object.

**Figure 1:**
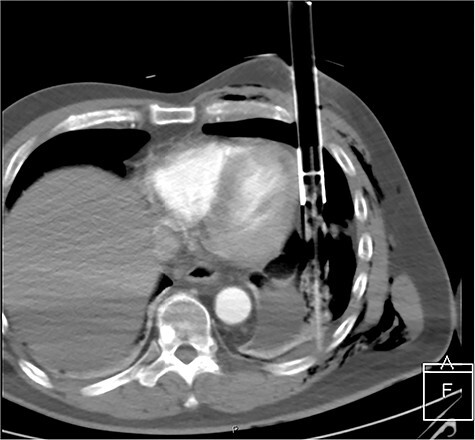
CT axial view of penetrating solar powered garden light demonstrating proximity of metal shaft to heart and the plastic tip resting against the posterior thorax.

**Figure 2:**
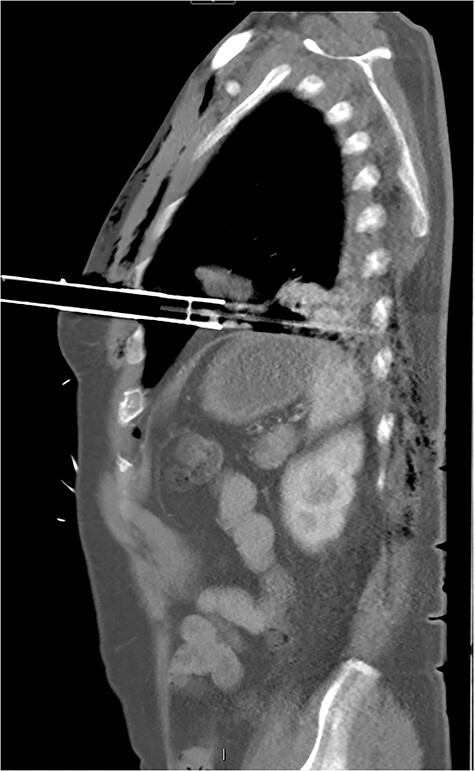
CT sagittal image showing depth of intrathoracic penetration by solar powered garden light.

**Figure 3:**
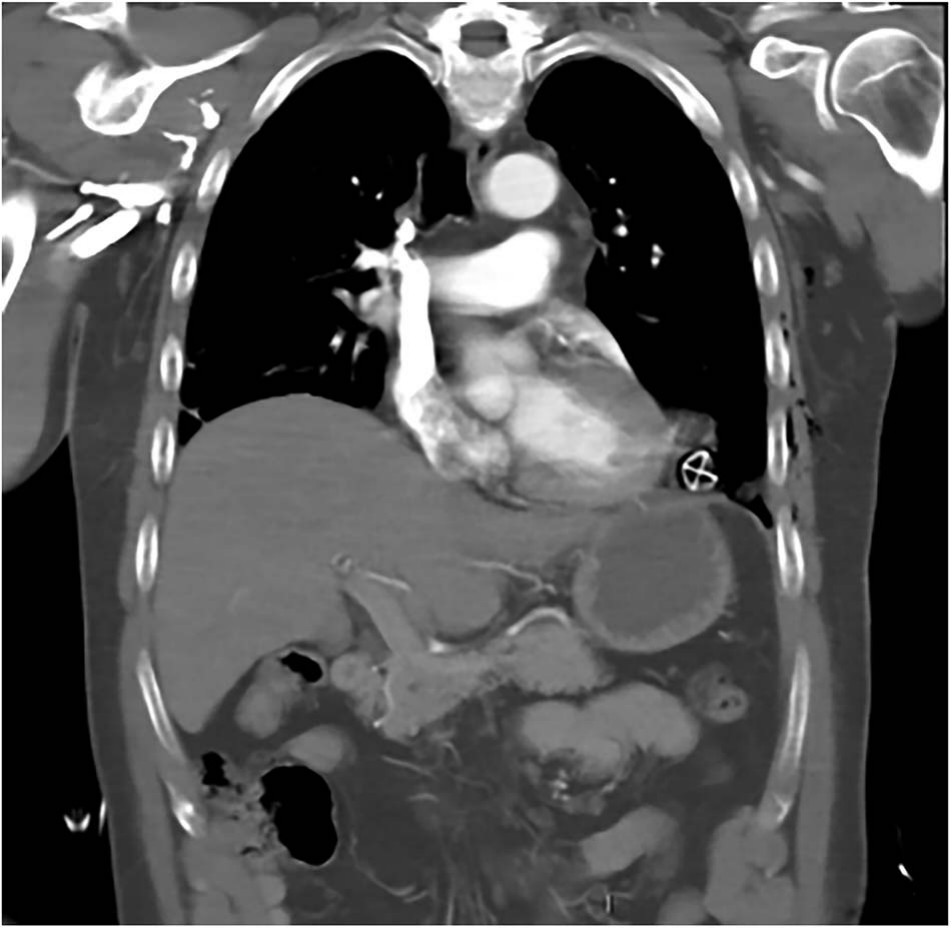
CT coronal image showing solar powered garden light proximity to diaphragm and cardiac border.

After primary survey and usual trauma imaging showed no air of fluid in either hemithorax, the patient underwent an emergent computed tomography (CT), which revealed a metal pole traversing the left hemithorax penetrating to the posterior chest wall at the level of the ninth rib. The metal pole was abutting the pericardium and was touching the posterior chest wall (Figs 1, 2 and 3).

The patient proceeded emergently to the operating theatre and underwent emergency posterolateral thoracotomy. This revealed that the plastic stake (connected to the metal rod) had created a small pericardial haematoma within pericardial fat, a 9 mm left hemi-diaphragmatic laceration with fat herniation in the thoracic cavity and perforated both the upper and lower lobes at multiple points. The thoracic cavity was contaminated with the patient’s skin and hair, as well as dirt and grass. The metal shaft and plastic stake were removed under direct vision and the injured lung parenchyma was managed with simple wedge resections. The diaphragmatic injury was repaired with 0 Vicryl. Thereafter, thoracic cavity was extensively washed out with 3 litres of warm normal saline and closed with apical and basal drains in situ. The entry wound was also washed out and closed in two layers. The patient was transferred to the intensive care unit and extubated the following day. The patient reported persecutory delusions and was diagnosed with an acute psychotic episode. This chest drains were removed after 3 days, and the patient received 5 days of broad-spectrum intravenous antibiotics. After this period, he was deemed medically fit for transfer to an acute inpatient psychiatric for further assessment and management.

## DISCUSSION

The management of a patient with chest trauma is best undertaken using the Advanced Trauma Life Support (ATLS) protocol. The primary survey should assess for the conditions that require immediate intervention, which are tension pneumothorax, open pneumothorax, flail chest, massive haemothorax and cardiac tamponade [1]. The immediate goals should be securing a patent airway, achieving oxygen saturations of ≥94%, gaining large bore intravenous access, providing fluids to support end organ function, conducting relevant haematological and radiological investigations, and the provision of spinal protective measures [2].

The frequency of penetrating thoracic injuries varies between geographical regions with rural and urban hospitals often having similar levels of mortality [3]. For patients who survive the initial injury, the most important predictor of mortality is distance or time to receiving adequate treatment [4]. This has been achieved, in part, by the generation of trauma referral networks where patients admitted to rural hospitals can be transferred to larger centres to receive specialist care [5].

An extensive search of the literature revealed no prior reported cases of penetrating thoracic trauma from solar powered garden lights. Regardless of the penetrating object, the initial management of chest trauma should be informed by the ATLS [1].

The management of retained foreign bodies follows similar ATLS principles but carry their own specific management challenges. They require careful assessment and surgical planning to be undertaken prior to the removal of a retained object, as unplanned extraction is associated with increased morbidity and mortality [6]. The removal of any retained foreign body that penetrates a cavity should be removed under direct vision.

Self-inflicted stab wounds are a well-recognized, but uncommon, method of suicide accounting for <2% of all suicides in Australia [7]. Analysis of patients who intentionally stabbed themselves reveals two distinct groups: the first is intoxicated young males who suffer from antisocial personality disorders without clear intent to end their lives; the second group includes those suffering from acute psychotic illnesses and are intentionally attempting to end their lives [8]. A significant number of patients who present post self-inflicted stabbing suffer from a psychotic illness. They are more likely to have injuries to the chest or abdomen as opposed to patients with other psychiatric disorders [9].
